# An area-based imaging biomarker for characterizing coronary artery stenosis with myocardial BOLD MRI

**DOI:** 10.1186/1532-429X-13-S1-O22

**Published:** 2011-02-02

**Authors:** Sotirios A Tsaftaris, Richard Tang, Xiangzhi Zhou, Debiao Li, Rohan Dharmakumar

**Affiliations:** 1 Northwestern University, Evanston, IL, USA; 2 Northwestern University, Chicago, IL, USA; 3 Cedars-Sinai Medical Center, Los Angeles, CA, USA

## Introduction

BOLD MRI may be used for detecting myocardial oxygenation changes secondary to coronary artery stenosis. However, current approaches for analyzing BOLD changes are suboptimal for detecting critical stenosis (reduction in perfusion reserve below 2:1).

## Purpose

To test the hypothesis that, ARREAS, an area-based statistical approach relying on the differences between rest and stress images, can characterize BOLD changes with exquisite sensitivity and specificity. This hypothesis was tested in a canine model.

## Methods

2D cine SSFP-based BOLD images were acquired in 9 dogs under rest, and adenosine stress with and without LCX stenosis (of varying grades) in a 1.5T scanner. Scan parameters: resolution=1.2x1.2x6mm^3^; flip-angle=90^o^; and TR/TE=6.2/3.1ms. Microsphere analysis was used to measure true perfusion. First-pass perfusion and late-enhancement scans were performed to visually confirm perfusion deficits and absence of infarction. Microsphere flow within each AHA segment was summed to obtain total flow per slice. *MFR*, defined as the ratio of flow between stress and rest was computed. End-systolic (ES) and end-diastolic (ED) images were identified and myocardial borders were traced. Myocardial pixel intensities from rest images were fitted to location-scaled t-distribution to estimate the location (*μ*) and scale (*σ*) parameters. *Affected-Fraction* (AF), defined as the ratio of the area of largest contiguous hypointense region (pixel intensity below *μ-σ*) divided by the total area of the myocardium, was computed for both stress (AF_STRESS_) and rest (AF_REST_) cases. *Ischemic-Extent* (IE), was defined/computed as IE=AF_STRESS_/AF_REST_. For comparison, mean signal intensities of AHA segments corresponding to the LCX territory were normalized by the mean intensity of the entire myocardium to obtain I_REST_ and I_STRESS_. *Segment-Intensity-Response* (SIR), was defined/computed as SIR=I_STRESS_/I_REST_. IE and SIR derived from ES and ED images were each regressed with MFR. ROC analysis was used to examine the diagnostic capacities of IE and SIR metrics to detect critical stenosis at ES and ED.

## Results

**Figure 1:**
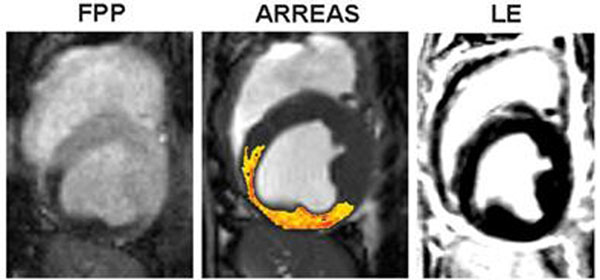
Relation between first-pass perfusion (FPP), myocardial BOLD image processed using ARREAS (Are-based biomaRker for chaRactErizing coronAry Stenosis), and late-enhancement (LE) images. FPP image obtained under adenosine stress with critical LCX stenosis and the corresponding BOLD image (processed with the ARREAS method matched to the trigger time of the FPP image) are shown for comparison. LE image acquired (at rest, prior to euthanization) at the same slice position and approximately the same trigger time, confirms the absence of any infarction. Note the close correspondence between the FPP and the BOLD image processed using ARREAS under the similar physiological conditions.

**Figure 2:**
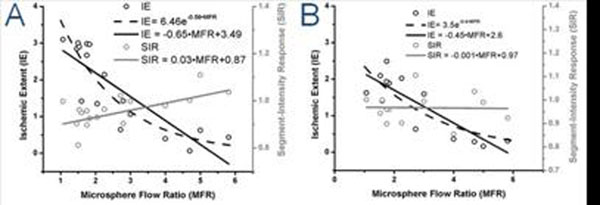
Scatter plots and firs of *Ischemic Extent* (IE, **left-hand y-axis**) or *Segment-Intensity Response* (SIR, **right-hand y-axis**) vs. Microsphere Flow Ratio (MFR, **x-axis**), derived from BOLD images acquired at end-systole (**A**) and end-diastole (**B**). IE values derived from end-systole BOLD images shows a stronger correlation to an exponential (*R^2^*=0.8) than to a linear function (*R^2^*=0.7) of MFR, while SIR shows weaker (linear) correlation with MFR (*R^2^*=0.5). IE values derived from end-diastole BOLD images shows and equivalent correlation with exponential and linear functions (*R^2^*=0.7) of MFR, while SIR shows no correlation to linear or exponential functions of MFR (*R^2^*~0). Statistical power at significance level 0.05 was almost 1 for all regressions. These results indicate that the myocardial BOLD effect is more reliably captured with a metric reflecting the size of the affected region (such as the propose IE) than with a metric reflecting mean intensity changes (such as the conventional SIR).

**Figure 3:**
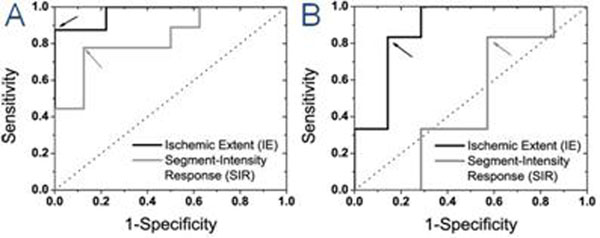
Receiver-Operating Characteristic (ROC) Analysis. ROC curves for *Ischemic Extent* (IE) and *Segment-Intensity Response* (SIR) metrics derived on the basis of end-systole (**A**) and end-diastole (**B**) BOLD images, for detecting critical stenosis (Microsphere Flow Ratio ≤2) are shown. For reference, the line of no discrimintation is also shown (dotted line). The similarity colored arrows point to the optimal operating cutodd points for each method. ROC analysis showed: for IE (ES or ED), sensitity (88% or 83%), specificity (100% or 86%)m and Area-Under-the-Curve (0.97 or 0.88); for SIR (ES or ED), sensitivity (77% or 83%), specificity (88% or 43%) and Area-Under-the-Curve (0.83 or 0.47). IE, derived using ARREAS, shows superior sensitivity and specificity compared to currently used approaches relying on the mean intensity of myocardial segments (SIR).

Fig. 1 shows a representative first-pass perfusion, ARREAS-processed BOLD, and late-enhancement images from a severe stenosis study. Fig. 2 illustrates scatter plots and fits between IE or SIR and MFR from 26 studies*.* Fig. 3 illustrates ROC curves.

## Conclusions

BOLD MRI is a compelling approach for evaluating myocardial oxygenation changes due to coronary stenosis. Compared to the conventional approach, ARREAS significantly increases the sensitivity and specificity for detecting BOLD changes; and offers the ability to quantify such changes on the basis of a metric that reflects the area of the myocardial territory affected by the stenosis.

